# Analysis of different therapeutic protocols for osteonecrosis of the jaw associated with oral and intravenous bisphpsphonates

**DOI:** 10.4317/medoral.21477

**Published:** 2016-12-06

**Authors:** Elena-Beatriz Bermúdez-Bejarano, María-Ángeles Serrera-Figallo, Aida Gutiérrez-Corrales, Manuel-María Romero-Ruiz, Raquel Castillo-de-Oyagüe, José-Luis Gutiérrez-Pérez, Guillermo Machuca-Portillo, Daniel Torres-Lagares

**Affiliations:** 1 Master’s Degree in Oral Surgery. School of Dentistry. University of Seville; 2 Associate Professor. Master’s in Integrated Dentistry and Patients with Special Diseases. School of Dentistry. University of Seville; 3 Department of Stomatology. School of Dentistry. Complutense University of Madrid; 4 Professor of Integrated. Dentistry and Patients with Special Diseases. School of Dentistry. University of Seville

## Abstract

**Introduction:**

Chemotherapy-associated osteonecrosis of the jaw caused by bisphosphonates is an exposure of necrotic bone with more than eight weeks of evolution that is attributable to bisphosphonates and no prior radiation therapy. Its etiopathogenesis remains unknown, although there are two hypotheses that may explain it: the drug’s mechanism of action, and the risk factors that can lead to osteonecrosis. There is a wide range of treatment options for managing chemotherapy-associated osteonecrosis of the jaw, from conservative treatments to surgical procedures of varying levels of invasiveness, which are sometimes supplemented with adjuvant therapies.

**Objectives:**

The objective of this article is to group the therapeutic options for osteonecrosis of the jaw (ONJ) into seven different protocols and to evaluate their effectiveness in relation to stage of ONJ.

**Material and Methods:**

A literature review was carried out in PubMed following the PRISMA criteria. A total of 47 were collected after compiling a series of variables that define ONJ, applied treatments, and the clinical results obtained.

**Results and Discussion:**

The 47 articles selected have a low to average estimated risk of bias and are of moderate to good quality. According to the data obtained, Protocol 3 (conservative treatment, clinical and radiological follow-up, minimally invasive surgical treatment, and adjuvant therapies) is the most favorable approach for ONJ lesions caused by oral bisphosphonates. For lesions caused by intravenous bisphosphonates, Protocol 2 (conservative treatment, clinical and radiological follow-up, minimally invasive surgical treatment, and no adjuvant therapies) is the best approach. When comparing the different stages of ONJ, Protocol 1 (conservative treatment, clinical and radiological follow-up) promotes better healing of Stage 1 ONJ lesions caused by orally administered bisphosphonates, and Protocol 3 is recommended for Stage II. For ONJ lesions attributable to intravenous bisphosphonates, Protocol 7 (conservative treatment, clinical and radiological follow-up, and adjuvant therapies) provides the best results in Stage 0; in Stages I, II, and III, Protocol 1 gives better results.

**Key words:**Bisphosphonates, bronj, therapeutic protocol, clinical result.

## Introduction

Bisphosphonates are stable, inorganic pyrophosphate analogs that are classified by their route of administration (oral or intravenous) and chemical composition (nitrogenous and non-nitrogenous). They are indicated for metabolic bone diseases (osteoporosis, osteogenesis imperfecta, Paget’s disease, etc.) or malignant hypercalcemia (multiple myeloma, cervical, lung, or mammary cancer, etc.). Many studies have proved their effectiveness in palliating bone and articular pain and in avoiding bone fractures, but due to their mechanism of action, in 2003, Marx warned of a complication: chemotherapy-associated osteonecrosis of the jaw (1).

Chemotherapy-associated osteonecrosis of the jaw refers to an exposure of necrotic bone with more than eight weeks of evolution that is attributable to bisphosphonates and no prior radiation therapy. Although its etiopathogenesis is unknown, two hypotheses could explain it: 1) the drug’s mechanism of action, which inhibits normal bone remodeling, impairs angiogenesis, increases the toxicity of soft tissues, and promotes dysfunctional modulation of the immune system; 2) the risk factors that trigger ONJ: local factors (oral surgery, prosthetic trauma, mandibular or palatal tori, ulcers, etc.), systemic and demographic factors (endocrine disruption [caused by obesity, diabetes, etc.], tobacco use, alcohol consumption, age, race, etc.), and genetic factors (cytochrome P450, nucleotide polymorphism [SNPs], etc.) (2).

ONJ encompasses a range of different stages. Depending on how advanced a stage is, the presence or absence of symptomatology, and/or whether there is any exposure of necrotic bone, there are different treatment alternatives indicated for management of chemotherapy-associated osteonecrosis of the jaw. These alternatives are divided into: 1) conservative treatment: antibiotics, analgesics, antiseptics, and antifungals; 2) minimally invasive surgical treatment (curettage or debridement of the exposed area, contouring of sharp bony edges, sequestrectomy with or without teeth involvement, etc.) or invasive surgical treatment (marginal and segmental resection with reconstruction of defective bone and soft tissues); 3) adjuvant therapies (PRP, laser, BMP, teriparatide therapy, ozone therapy, oxygen therapy, and photodynamic therapy). Adjuvant therapies will promote to a greater or lesser degree the correct healing of bone and soft tissues (3).

As chemotherapy-associated osteonecrosis of the jaw is a complex condition, it is problematic not only for dentists but also for oral and maxillofacial surgeons; while there are many different treatment options, it not always clear which approach is the best for any given case.

While the best therapeutic option is simply to prevent ONJ from occurring in the first place, once it is diagnosed, treatment focuses on healing as well as offering the patient a better quality of life, focusing not only on the lesions but also on the complications they may cause.

This article seeks to use published evidence to identify the different therapeutic options that can be applied to osteonecrosis of the jaw and assess their effectiveness according to the stage of the disease in which they are applied.

## Material and Methods

A review of the literature published in PubMed was carried out from January 2002 (the year in which osteonecrosis of the jaw was first described) to September 2015 using the following keywords: “osteonecrosis jaw AND surgical approach,” “osteonecrosis jaw AND laser therapy,” “osteonecrosis jaw AND hyperbaric oxygen,” “osteonecrosis jaw AND PRP,” “antibiotic prophylaxis AND osteonecrosis,” “bisphosphonates AND osteonecrosis AND dental management,” “osteonecrosis jaw AND protocol bisphosphonates,” “osteonecrosis jaw AND conservative protocol,” and “osteonecrosis jaw AND surgical protocol.”

Searches with these keywords returned 61, 31, 34, 9, 31, 133, 55, 12, and 44 results, respectively. The following inclusion and exclusion criteria were applied.

The inclusion criteria were: 1) academic publications written in English that use the clinical diagnosis of osteonecrosis of the jaw as established by the AAOMS and the ASBMR (4); 2) the following types of studies: clinical trials and case series with more than five patients; 3) articles that identify the route of administration of bisphosphonates, the risks factors for development of ONJ, the treatment protocol chosen, and its results.

The exclusion criteria were: 1) articles that did not meet the inclusion criteria; 2) articles unrelated to the research topic; 3) articles with less than five human patients, letters to the editor, and expert opinions.

The automatic search was complemented with a manual verification of all bibliographic references from the collected articles, choosing any additional articles that were relevant to the present study and that met the inclusion and exclusion criteria used.

A dual approach was used to assess overall quality, with each article being evaluated for both risk of bias and the total number of variables collected.  Table 1 shows the criteria used to gauge the risk of bias. Thus, if an article has two or three zeros, the risk of uncontrolled bias is low; with four zeros, the risk of uncontrolled bias is medium, and if an article has more than four zeros, the uncontrolled risk of bias is high, and the article will consequently be eliminated from the study.

**Table 1:**
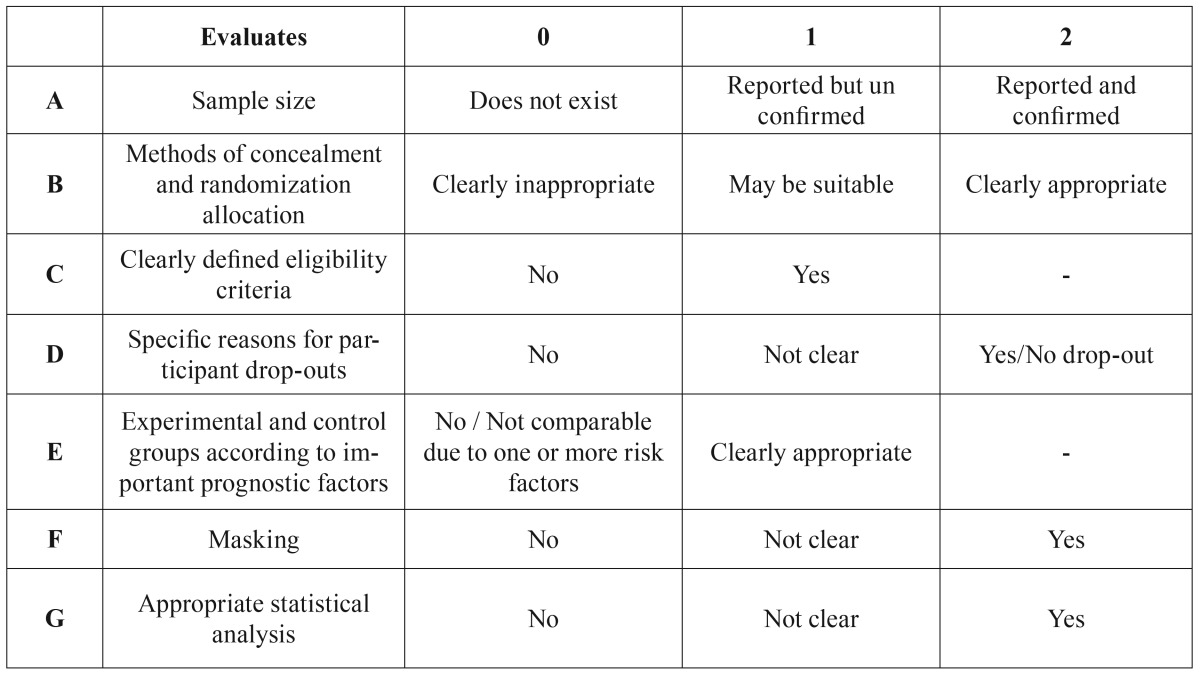
Risk of bias control assessment.

To evaluate the suitability of the variables provided in each article, the total number of collected variables was assessed using a modification suggested by the AAOMS and ASBMR (4). The articles were evaluated using 12 parameters involving ONJ and its stage of advancement, study design, number of patients, average age, type and route of administration of the bisphosphonate, location of the ONJ, cause for therapy with bisphosphonates, trigger factor/s, risk factors for osteonecrosis, the treatment applied, and the results obtained with this treatment. Depending on the information collected in each article, the publication’s quality was classified as good (10-12 variables provided), moderate (5-9 variables provided) or poor (1-4 variables provided).

To reach the proposed objectives, after grouping all the aforementioned variables by choice of treatment (conservative treatment, minimally invasive surgical treatment, and invasive surgical treatment), defining parameters of the clinical approach for each option were collected: 1) conservative treatment: type of mouthwash, intraoral gels, antifungal drugs, analgesics, and antibiotics used as well as the protocol for discontinuing use, if any; 2) minimally invasive surgical treatment: type of conservative treatment, type of minimally invasive surgical technique used, and whether it was accompanied by any adjuvant therapies; 3) invasive surgical treatment: type of conservative treatment, type of invasive surgical treatment used, and whether or not an adjuvant therapy was used.

All the data were grouped into seven different protocols, with the results obtained in ONJ lesions also being studied after the application of these protocols. The seven protocols are: 1) Protocol 1: conservative treatment, clinical and radiological follow-up; 2) Protocol 2: conservative treatment, clinical and radiological follow-up, minimally invasive surgical treatment without adjuvant therapies; 3) Protocol 3: conservative treatment, clinical and radiological follow-up, minimally invasive surgical treatment, and adjuvant therapies; 4) Protocol 4: conservative treatment, clinical and radiological follow-up, invasive surgical treatment without adjuvant therapies; 5) Protocol 5: conservative treatment, clinical and radiological follow-up, invasive surgical treatment, and adjuvant therapies; 6) Protocol 6: adjuvant therapies, clinical and radiological follow-up; 7) Protocol 7: conservative treatment, clinical and radiological follow-up, and adjuvant therapies.

A number of synonyms have been found in the selected articles regarding the type of clinical results obtained after treatment of ONJ lesions. To simplify the results found, a general name has been used for these: complete healing, partial healing (lesion with remission, lesion with moderate improvement, stable lesion, and non-recurrent lesion) and lesions that become worse (recurrent lesion, uncontrolled lesion, worsening lesion, lesion that fails to heal, and progressive lesion). These three results will be used to evaluate the effectiveness of each protocol.

## Results

**Figure 1:**
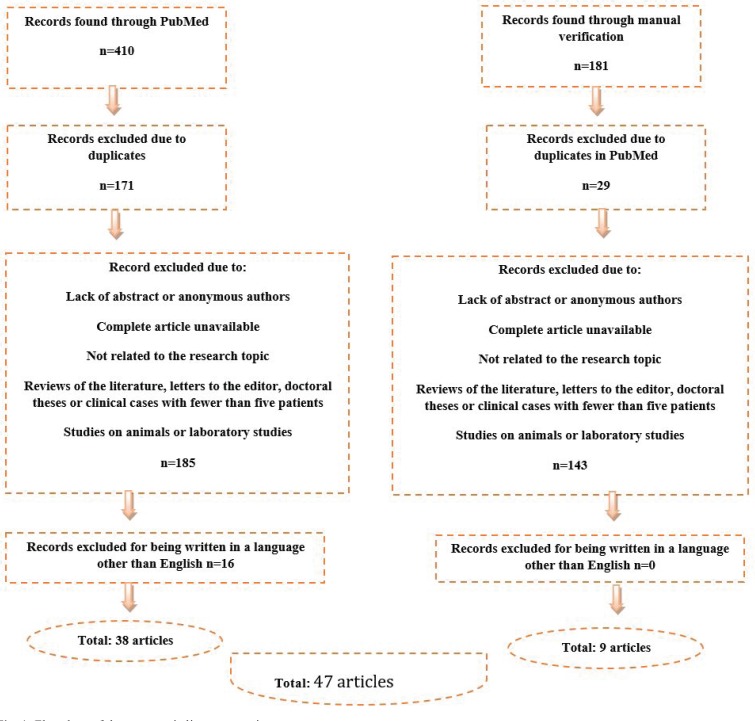
Flowchart of the systematic literature review.

The initial search in PubMed yielded 410 results, with an additional 181 articles collected during the manual search of bibliographic references (Fig. 1).

The 47 articles selected have a low to average estimated risk of bias and are of moderate to good quality. These evaluations can be seen in  Table 2,Table 3 and 3 continue.

**Table 2:**
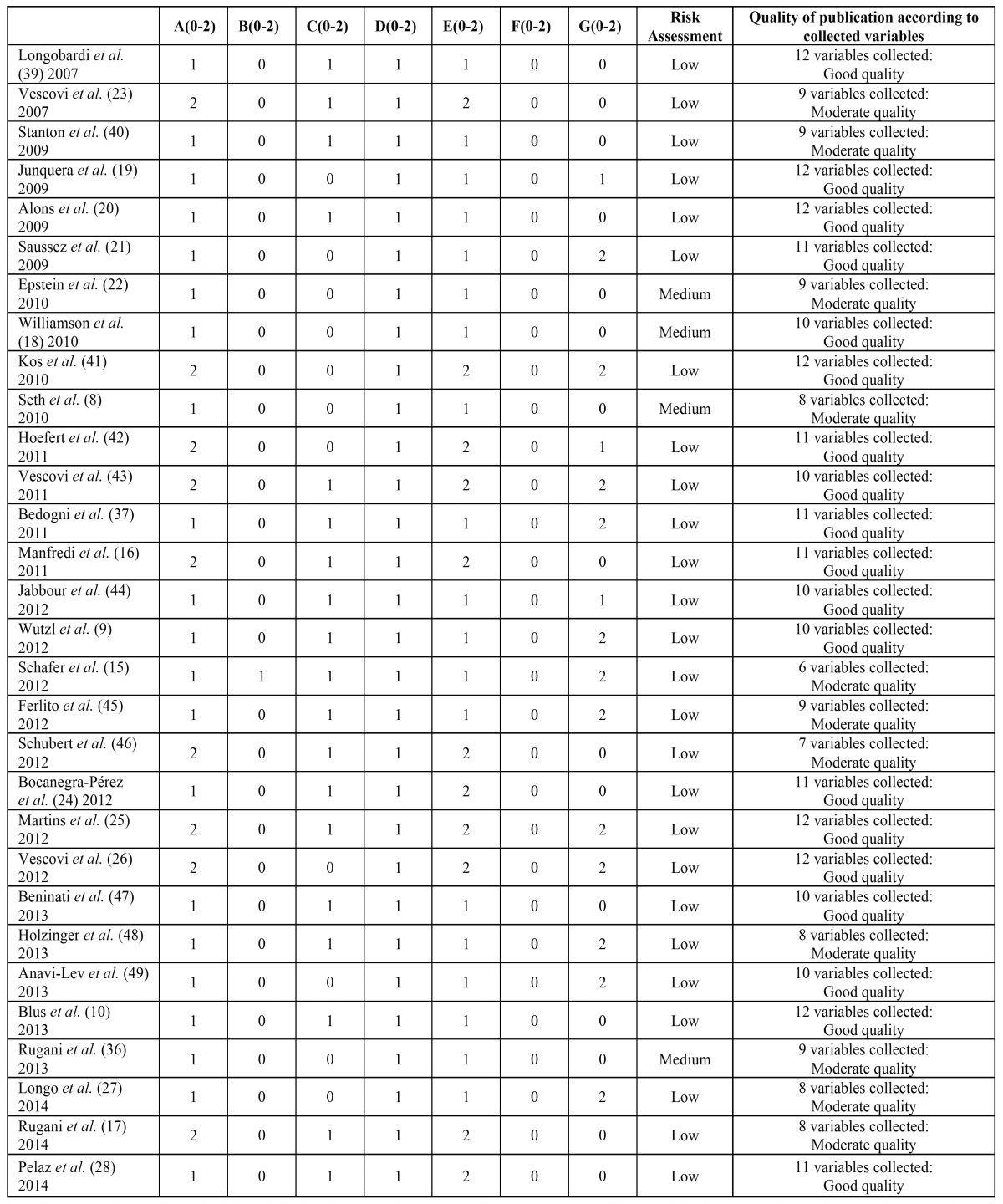
Evaluation of risk of bias and usefulness of the selected publications using oral bisphosphonates.

**Table 3:**
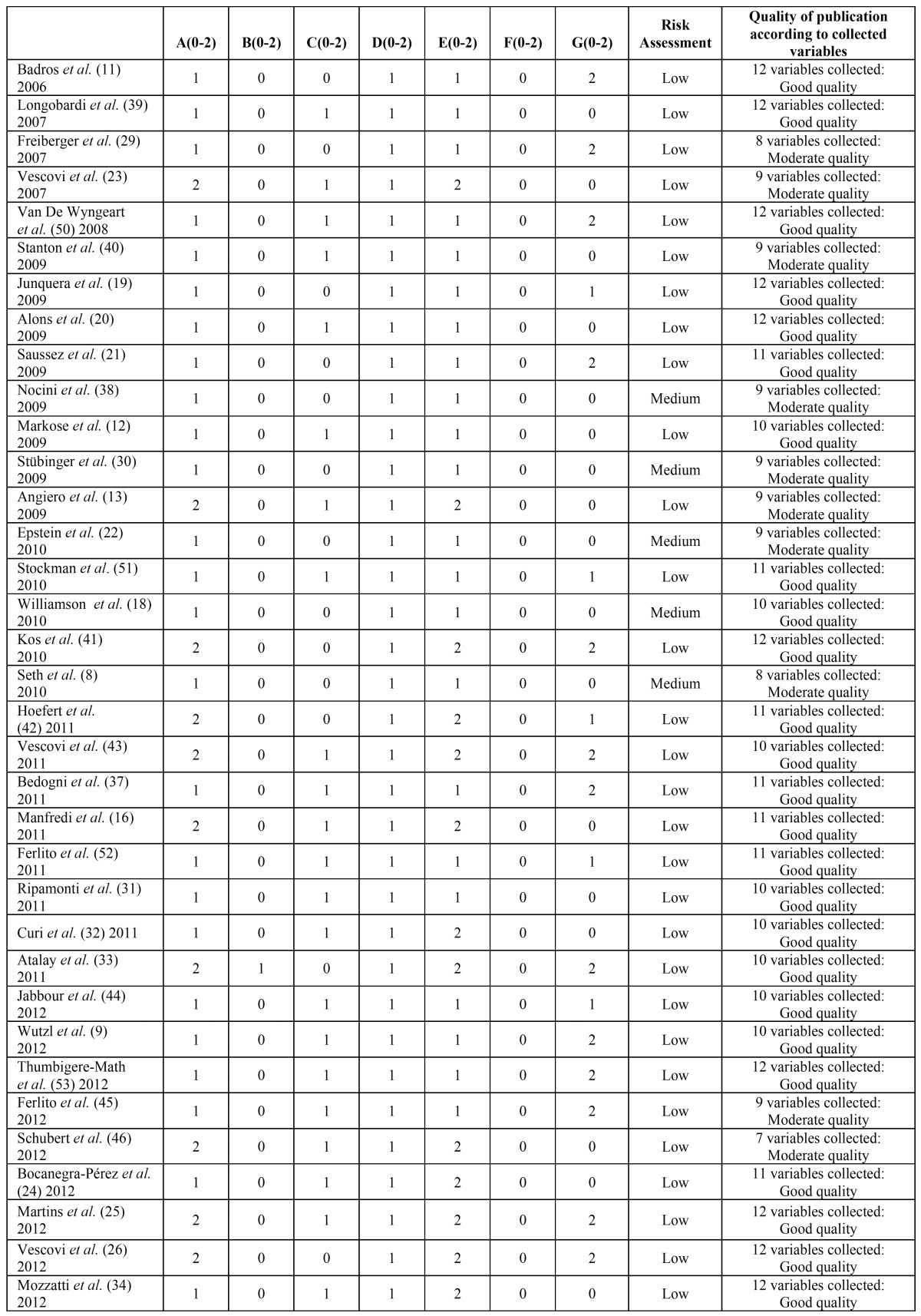
Evaluation of risk of bias and usefulness of the selected publications using intravenous bisphosphonates.

**Table 3 continue:**
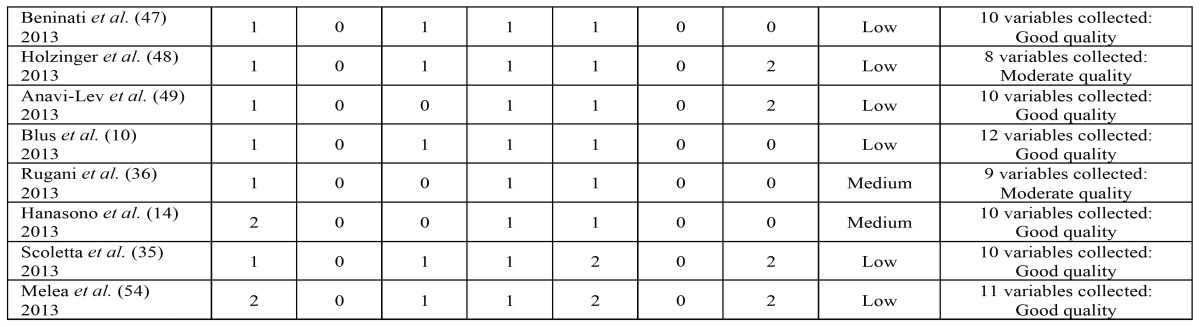
Evaluation of risk of bias and usefulness of the selected publications using intravenous bisphosphonates.

After selecting the articles, an initial analysis of each treatment option assessed the 12 variables of ONJ, as well as the seven aforementioned protocols, to produce the tables in which the overall results of each protocol for ONJ lesions caused by oral bisphosphonates ( Table 4) and intravenous bisphosphonates ( Table 5) are detailed.

**Table 4:**
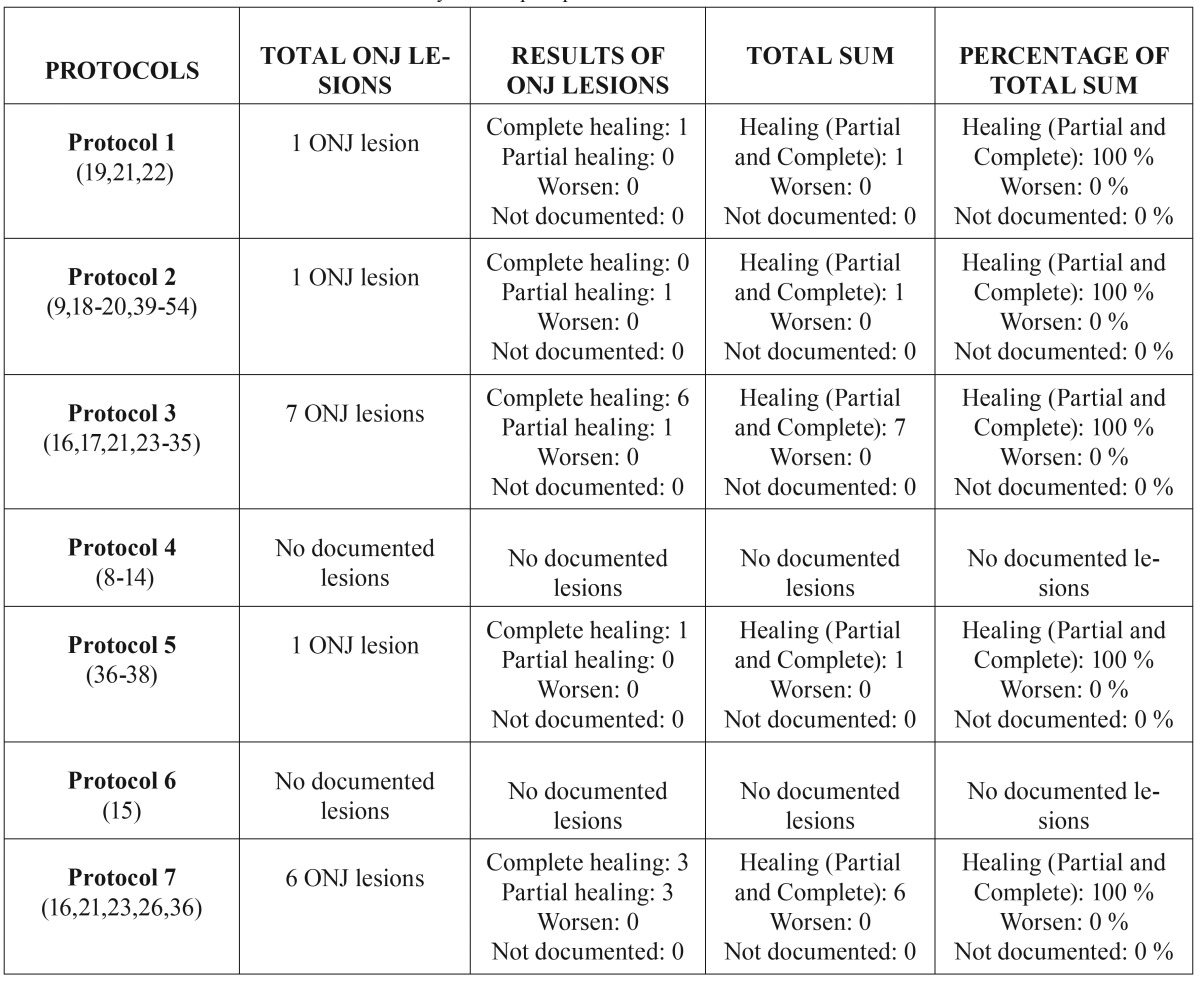
Overall results of ONJ lesions caused by oral bisphosphonates.

**Table 5:**
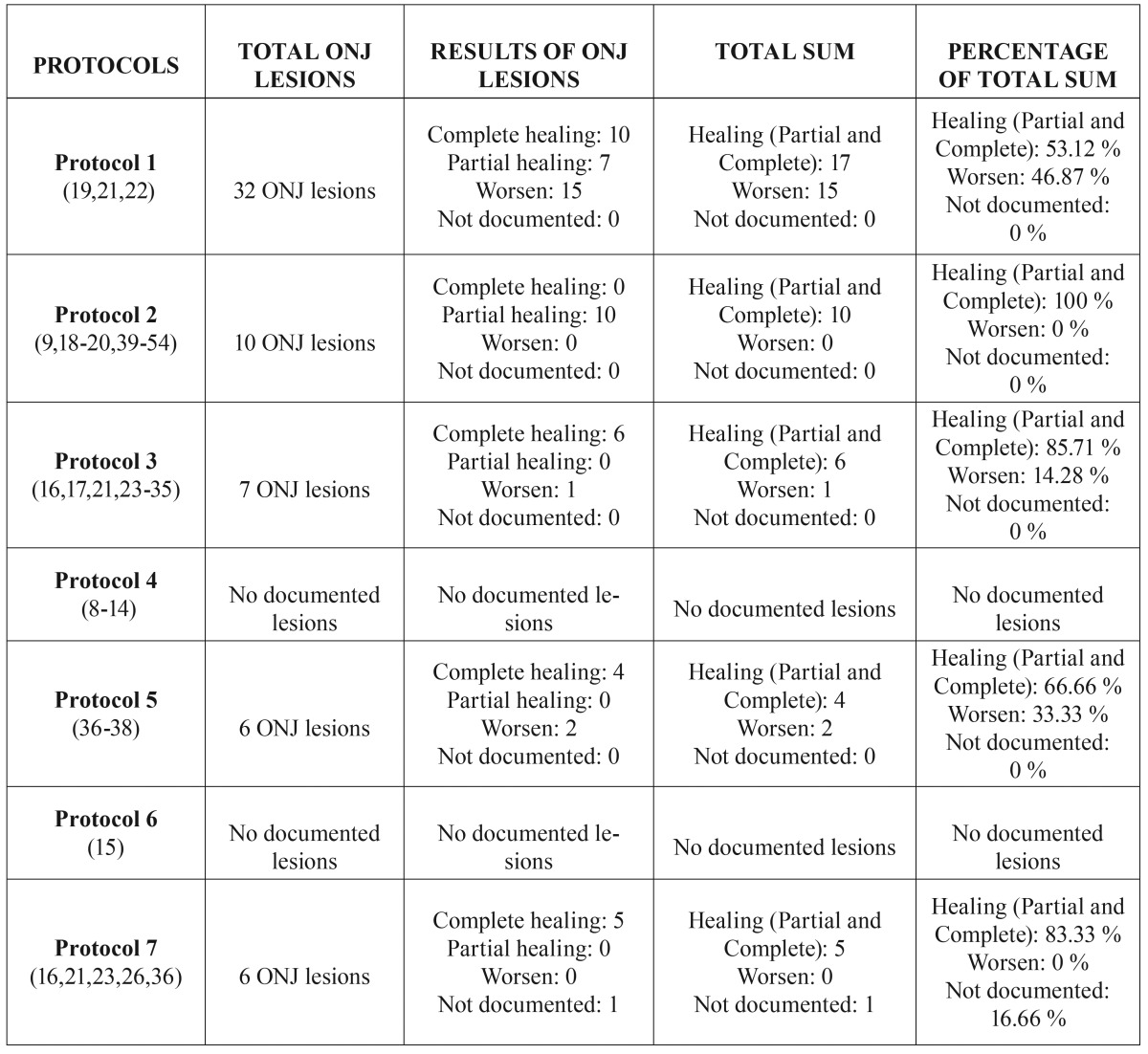
Overall results of ONJ lesions caused by intravenous bisphosphonates.

Subsequently, the data were analyzed and presented according to stage of advancement of the lesion and the protocol applied; this analysis can be seen in  Table 6 and 6 continue (oral bisphosphonates) and  Table 7 and 7 continue (intravenous bisphosphonates).

**Table 6:**
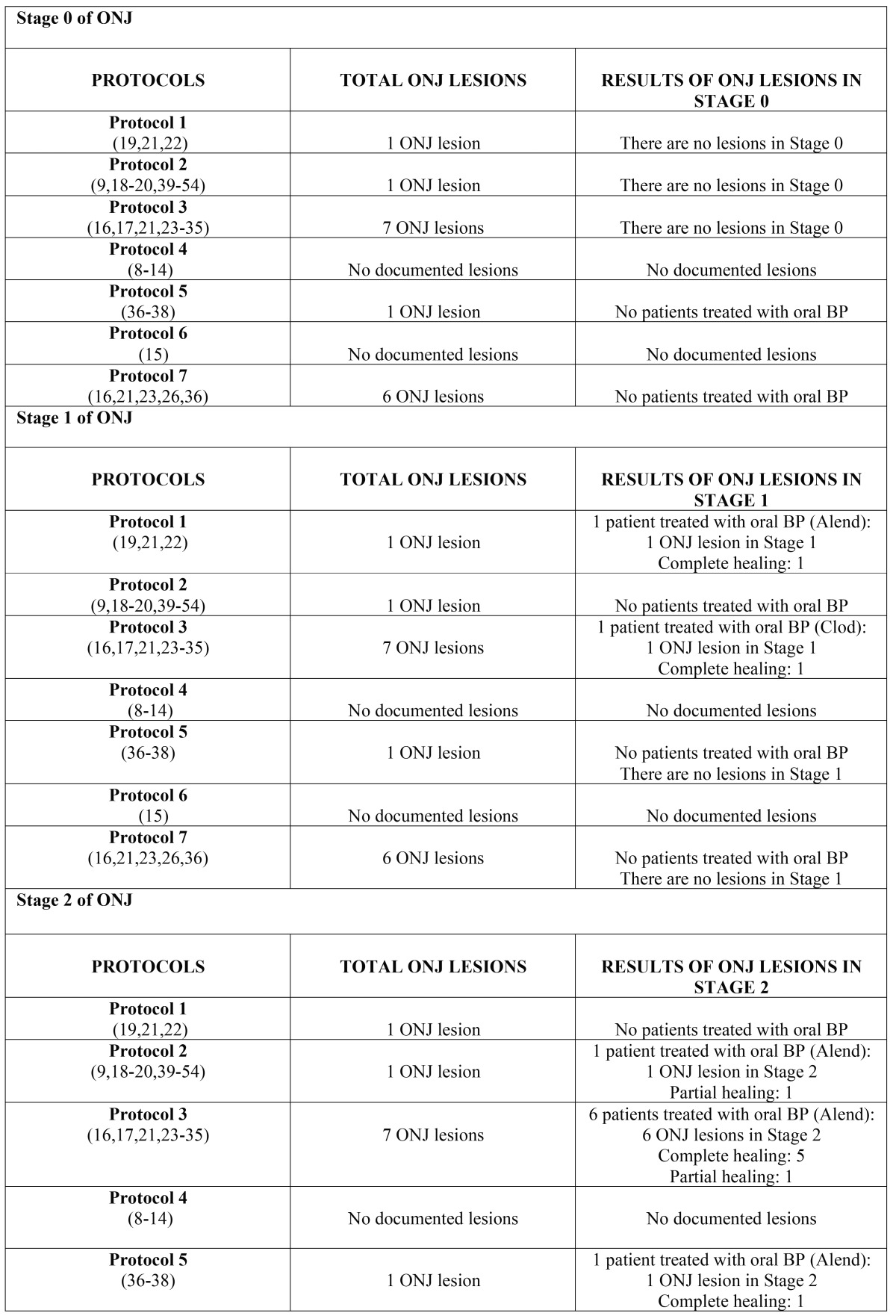
Results of lesions according to protocol and stage of ONJ caused by oral bisphosphonates.

**Table 6 continue:**
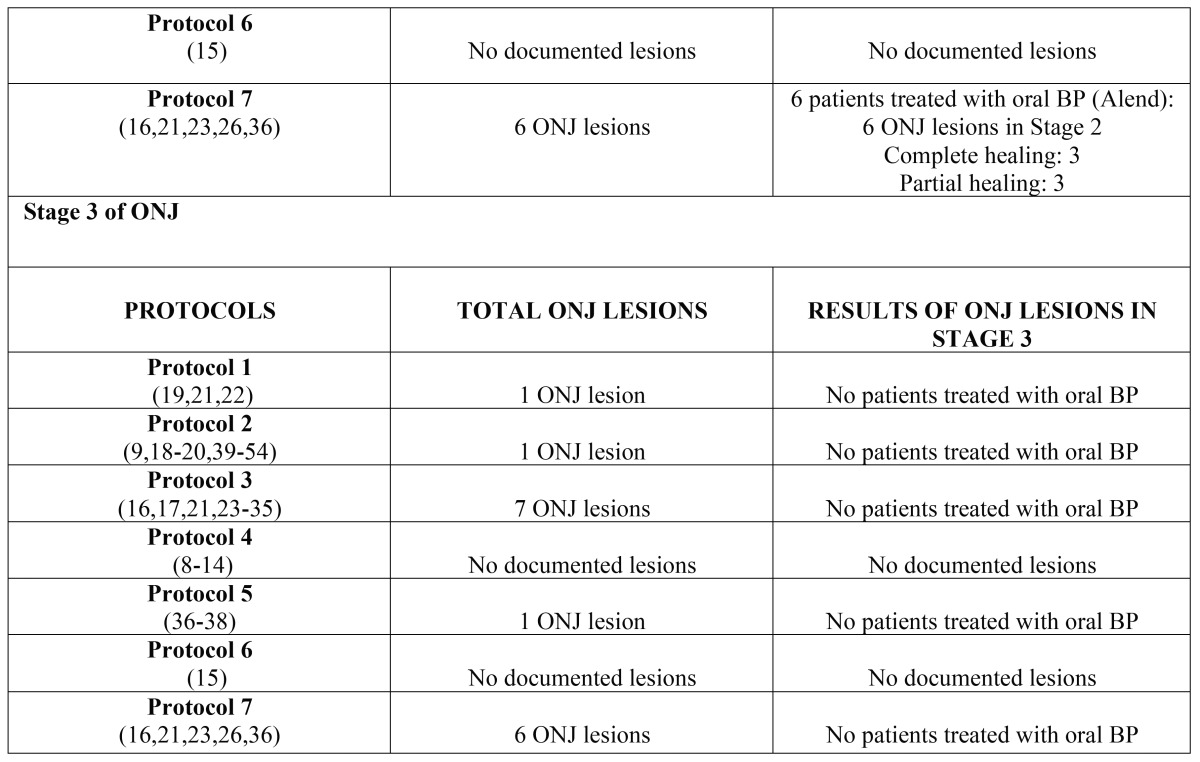
Results of lesions according to protocol and stage of ONJ caused by oral bisphosphonates.

**Table 7:**
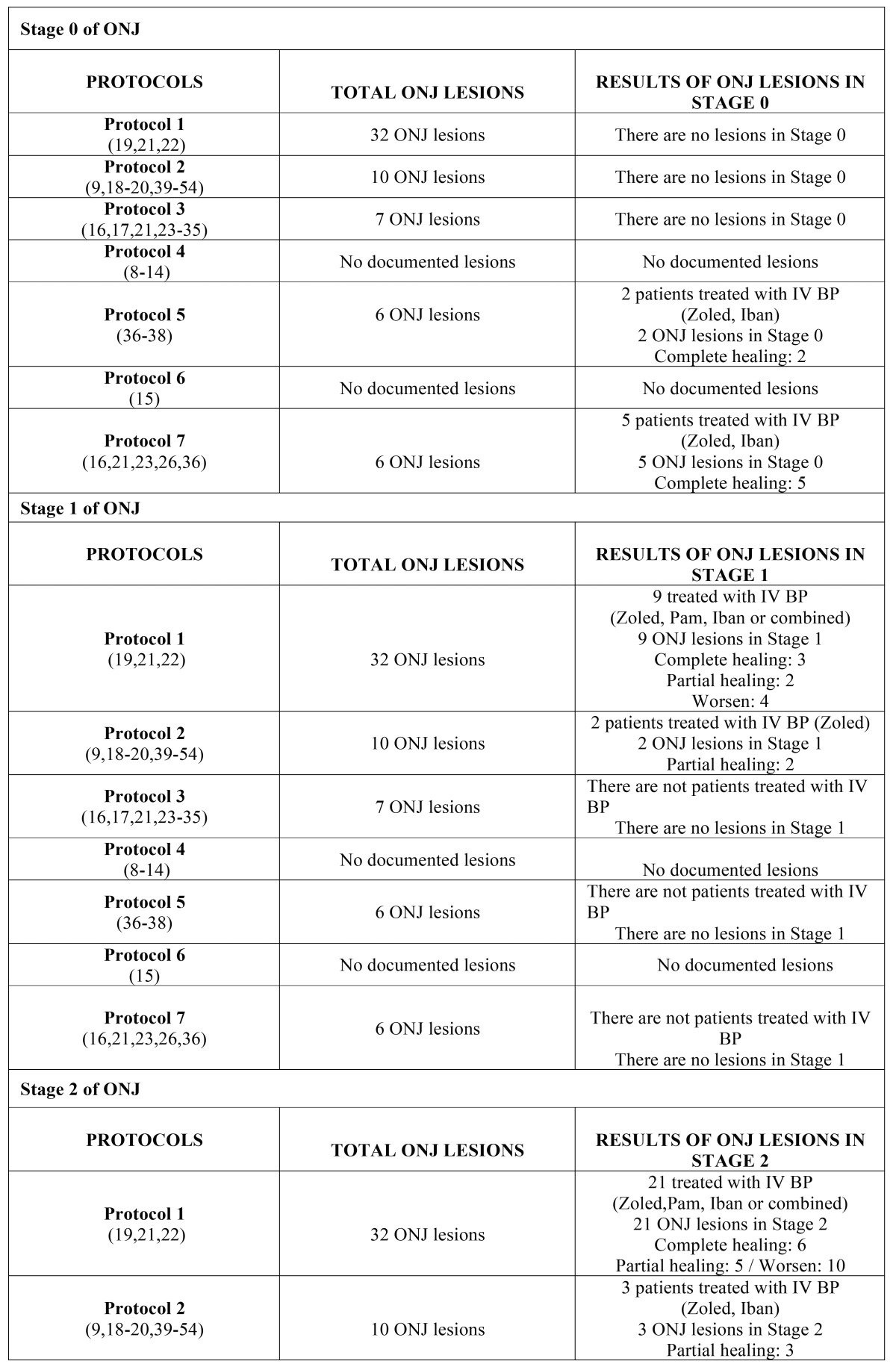
Results of lesions according to protocol and stage of ONJ caused by intravenous bisphosphonates.

**Table 7 continue:**
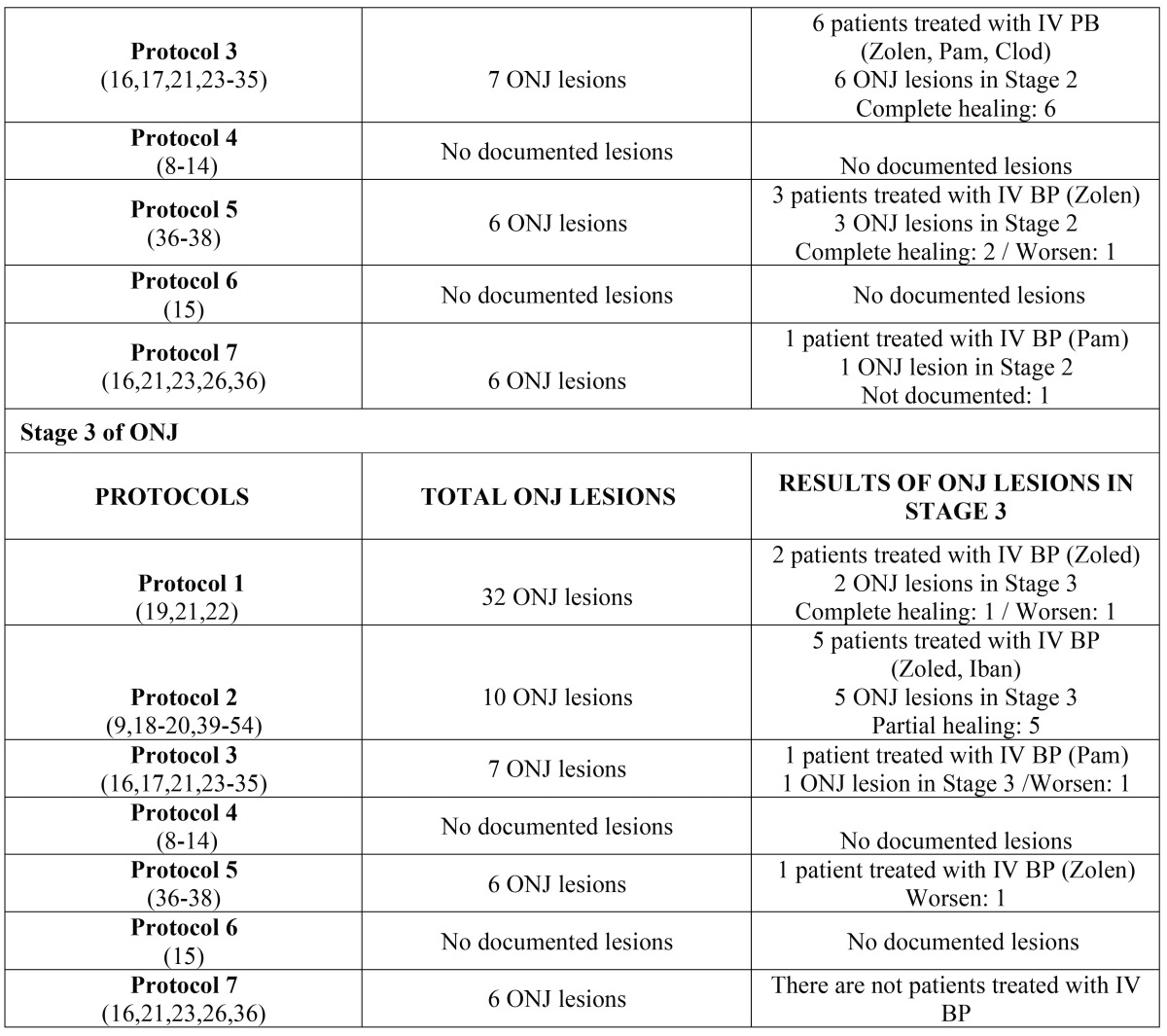
Results of lesions according to protocol and stage of ONJ caused by intravenous bisphosphonates.

## Discussion

After carrying out the bibliographic search, there appears to be a substantial amount of heterogeneity among the mentioned techniques and great controversy over which protocols are the best, with various results in the effective management of ONJ found (5-7).

The results of the seven evaluated protocols on lesions produced by osteonecrosis of the jaw can be used to predict which protocol will obtain better or worse results, both overall and in each individual stage (differentiating as well between the various routes of administration employed).

With regard to the overall effectiveness of the seven protocols in the treatment of ONJ lesions caused by oral bisphosphonates, it is clear that: 1) the partial or complete healing of ONJ lesions for each protocol, from most to least by percentage of ONJ cured, is: Protocol 3 (100%) > Protocol 7 (100%) > Protocol 1 (100%) Protocol 2 (100%) > Protocol 5 (100%); 2) no worsening of ONJ lesions was observed in any of these protocols.

Two of the protocols cannot be evaluated for either healing (partial and/or complete) or worsening of ONJ lesions due to different reasons: in Protocol 4 (8-14), the articles do not report the effect on ONJ lesions with respect to stage of severity, and in Protocol 6 (15), none of the articles describe the effect on ONJ lesions.

According to the results obtained, the protocols that obtain the best healing (partial or complete) of ONJ lesions are Protocols 3 and 7, with 100% of lesions healed (whether partial or complete). Although 100% of lesions were healed in all protocols except those that did not mention these results, the descending order mentioned above still applies, as Protocol 3 and 7 have seven and six lesions, respectively, and the remaining protocols (Protocols 1, 2, and 5) have only one.

However, as the studied articles had few ONJ lesions secondary to oral bisphosphonates, care should be taken when drawing firm conclusions from these results.

Focusing on the protocol this study found to be the best, Protocol 3 cures 100% of lesions, which coincides with the findings of other studies found in the literature that reference 89.5% to 91% healing of oral mucosa (16,17). This may be due to the minimally invasive surgical treatment (curettage or debridement of the exposed site, contouring, sequestrectomy with or without teeth involvement, etc.) used in this protocol, with these being the most common surgical techniques used in the management of chemotherapy-associated osteonecrosis of the jaw or refractory ONJ (18). In addition, this protocol is associated with other strategies such as conservative treatment (19) and adjuvant therapies (17), resulting in synergistic disease management. Nevertheless, the protocol’s prognosis may vary according to the stage and type of ONJ lesion, and it is not indicated for patients at risk of ONJ or in its early stages. This aspect will be discussed later.

Regarding the overall effectiveness of the seven protocols observed in the treatment of ONJ lesions caused by intravenous bisphosphonates, it is clear that: 1) the healing (partial or complete) of ONJ lesions for each protocol, following a sequential and descending order by percentage of healed ONJ, would be: Protocol 2, (100%) > Protocol 3 (85.71%) > Protocol 7 (83.33%) > Protocol 5 (66.66%) > Protocol 1 (53.12%); 2) the worsening of ONJ lesions for each protocol in sequential and descending order, by percentage of ONJ lesions that got worse, is: Protocol 1 (46.87%) > Protocol 5 (33.33%) > Protocol 3 (14,28%).

Two of the protocols cannot be evaluated for either healing (partial and complete) or worsening of ONJ lesions due to different reasons: 1) in Protocol 4 (8-14), none of the articles give results on ONJ lesions according to their stage of severity; and 2) in Protocol 6 (15), none of the studies describe the effects on ONJ lesions.

According to the results obtained, the protocols that obtain the best healing (partial or complete) of ONJ lesions are Protocols 2, 3, and 7, with 100%, 85%, 71%, and 83.33%, of lesions healed (whether partial or complete), respectively. With regard to worsening lesions, Protocol 1 had the highest number of worsened lesions, at 46.87%, and Protocol 5 had a rate of 33.33%.

The protocol with best results, Protocol 2, achieves a 100% lesion healing rate, comparable with the results of other published studies in which healing of oral mucosa was seen in 90% of the patients (20); however, not all studies obtained such results, with some only obtaining 58.5% of completely covered mucosa (9). This protocol is based on conservative treatment and minimally invasive surgical techniques, as previously seen in Protocol 3, with the only difference between Protocols 2 and 3 being the use of adjuvant therapies in the latter. As a result, the second best results were seen from Protocol 3, with 85.71% of lesions healed. However, as discussed below, its prognosis may vary according to the stage of the ONJ lesion.

Regarding the effectiveness of the results obtained using the seven protocols in the treatment of ONJ lesions caused by oral bisphosphonates, as evaluated in each of the different stages, it can be indicated that: 1) in Stage 0, no ONJ lesions were treated with the mentioned protocols in patients receiving orally administered bisphosphonates; 2) in Stage I (19,21,22), both Protocol 1 and Protocol 3 (16,17,21,23-35) obtained the best results; 3) in Stage II, the protocol with the best rate of healing is Protocol 3, (16,17,21,23-35) followed by Protocols 7, (16,21,23,26,36) 5, (36-38) y 2; (9,18-20,39-54) 4) in Stage III, no ONJ lesions were treated with the mentioned protocols in patients taking oral bisphosphonates.

In both Stage 0 and Stage III, there no ONJ lesions caused by oral bisphosphonates were observed; therefore, it is impossible to know which protocol obtains the best results. Consequently, Stages I and II can be assessed. In Stage I, the best results were seen in Protocols 1 and 3, and these protocols were the only ones in which lesions were recorded as completely cured, with one lesion identified per protocol. In Stage II, the protocol with the best results is Protocol 3, followed by Protocol 7. However, due to the limited number of lesions observed in each stage and treatment protocol for ONJ lesions caused by oral bisphosphonates, it would not be realistic to establish a valid uniform approach. It is true that the results obtained in Stage II could potentially be discussed further, with Protocol 3 being a therapeutic alternative with optimal results.

In regards to the effectiveness of the results obtained by the seven protocols in the treatment of each of the stages of ONJ lesions caused by intravenous bisphosphonates, it can be indicated that: 1) in Stage 0, Protocol 7 had the best results (16,21,23,26,36), followed by Protocol 5;(36-38) 2) in stage I, the best protocol was Protocol 1 (36-38), followed by Protocol 2; (9,18-20,39-54) 3) in Stage 2, the best protocol by rate of healing is Protocol 1, (19,21,22) followed by Protocols 3, (16,17,21,23-35) 5, (36-38) and 2; (9,18-20,39-54) 4) in Stage III, Protocol 1 had the best results (19,21,22), followed by Protocol 2 (9,18-20,39-54).

In stage 0, Protocol 7 obtained the best results, with five ONJ lesions treated with this protocol and 100% of the lesions cured completely. This may be because in early stages with unspecific radiological and clinical symptomatology and without necrotic bone exposure, the clinical and radiological follow-up, conservative treatment, and use of adjuvant therapies can be enough to keep the lesion stable so it does not progress and to promote remission of symptoms. These results match those found in the literature, as for example the study carried out by Rugani, (17) who found that a combination of oral mouthwashes, medical treatment and photodynamic therapy can promote secondary granulation and the formation of new scar mucosa. This combination would avoid more aggressive therapies and more advanced stages of ONJ, thereby achieving optimal results.

In ONJ Stages I, II and III, Protocol I obtained the best results, followed by Protocol 2 in Stages I and II, and Protocol 3 in Stage III. The rates of healing of ONJ lesions using Protocol 1 in ONJ Stages I, II and III are 55.55%, 52.38%, and 100%, respectively. These results match those found in the literature (around 57% of cured lesions and/or healed). (21) However, the obtained results show only Stage III lesion treated with Protocol 1, which was fully cured; therefore it is not especially substantial evidence. This should be taken into account when choosing between Protocols 1 and 3.

With regard to Protocol 1 therapy for Stages I and II, five out of nine lesions were cured, either partially or completely. If all protocols are included, in Stage I and Stage II, 11 out of 21 lesions were cured, either partially or completely. In these two stages (I and II), Protocol 1 can be identified as a potentially effective therapeutic tool.

Returning to discussion of the overall results, Protocol 1 paradoxically saw greater worsening of ONJ lesions overall, but for some stages, it obtained more partial and complete healing. During the early stages of the disease, a conservative approach in the treatment of chemotherapy-associated osteonecrosis of the jaw, which prioritizes the etiological treatment of symptomatology (analgesics, antibiotics, antifungals, mouthwashes, and treatment discontinuation) alongside clinical and radiological follow-up, is usually the first step in ONJ management because it promotes promising results, given the complexity of accurate and successful treatment of ONJ.

However, the effectiveness of each treatment and protocol may vary according to the stage ONJ (early or advanced), developed symptomatology, type of bony sequestrum, patient characteristics (medical and dental history), and different risk factors associated with ONJ. It is important to control all of these parameters in order to keep the disease from progressing to advanced stages (1-4).

While there are various different protocols for the management of ONJ, the best way to fight the disease is by preventing it, as this is the most ideal stage with guaranteed success, thereby improving quality of life in patients treated with bisphosphonates.

To conclude, it is clear that: 1) There are different therapeutic options for managing osteonecrosis of the jaw, such as conservative treatment, minimally invasive surgical treatment, invasive surgical treatment, and adjuvant therapies, which have been grouped in seven different protocols for the purposes of the present study. 2) Due to the small number of ONJ lesions caused by oral bisphosphonates, it is not possible to establish relevant criteria, hence there is a need for more studies with a higher number of ONJ lesions caused by orally administered bisphosphonates before reaching firm conclusions. 3) According to the seven grouped protocols and their obtained results, it can be stated that the best approach for ONJ lesions caused by oral bisphosphonates is Protocol 3, while the best option for lesions caused by intravenous bisphosphonates is Protocol 2. 4) According to the seven grouped protocols and their obtained results in each stage of ONJ, the protocol that resulted in better healing of Stage 1 ONJ lesions caused by oral bisphosphonates is Protocol 1, and Protocol 3 in Stage II (in Stages 0 and III, the consulted data does not detail any ONJ lesions caused by oral bisphosphonates). 5) Regarding ONJ lesions secondary to intravenous bisphosphonates, the protocol that obtained the best results in Stage 0 is Protocol 7, and in Stages I, II, and II, Protocol 1 is the best choice.
